# *Chlamydia trachomatis* Infection Impairs MHC-I Intracellular Trafficking and Antigen Cross-Presentation by Dendritic Cells

**DOI:** 10.3389/fimmu.2021.662096

**Published:** 2021-04-15

**Authors:** Diego Del Balzo, Anahí Capmany, Ignacio Cebrian, María Teresa Damiani

**Affiliations:** ^1^ Biochemistry and Immunity Laboratory, School of Medicine, University of Cuyo, IMBECU-CONICET, Centro Universitario, Mendoza, Argentina; ^2^ Instituto de Histología y Embriología de Mendoza (IHEM)-CONICET, Facultad de Ciencias Médicas, Universidad Nacional de Cuyo, Mendoza, Argentina

**Keywords:** cross-presentation, MHC-I recycling, *Chlamydia trachomatis*, dendritic cells, endocytic recycling compartment

## Abstract

During cross-presentation, exogenous antigens (i.e. intracellular pathogens or tumor cells) are internalized and processed within the endocytic system and also by the proteasome in the cytosol. Then, antigenic peptides are associated with Major Histocompatibility Complex (MHC) class I molecules and these complexes transit to the plasma membrane in order to trigger cytotoxic immune responses through the activation of CD8+ T lymphocytes. Dendritic cells (DCs) are particularly adapted to achieve efficient antigen cross-presentation and their endocytic network displays important roles during this process, including a sophisticated MHC-I transport dependent on recycling compartments. In this study, we show that *C. trachomatis*, an obligate intracellular pathogen that exhibits multiple strategies to evade the immune system, is able to induce productive infections in the murine DC line JAWS-II. Our results show that when *C. trachomatis* infects these cells, the bacteria-containing vacuole strongly recruits host cell recycling vesicles, but no other endosomal compartments. Furthermore, we found that chlamydial infection causes significant alterations of MHC-I trafficking in JAWS-II DCs: reduced levels of MHC-I expression at the cell surface, disruption of the perinuclear MHC-I intracellular pool, and impairment of MHC-I endocytic recycling to the plasma membrane. We observed that all these modifications lead to a hampered cross-presentation ability of soluble and particulate antigens by JAWS-II DCs and primary bone marrow-derived DCs. In summary, our findings provide substantial evidence that *C. trachomatis* hijacks the DC endocytic recycling system, causing detrimental changes on MHC-I intracellular transport, which are relevant for competent antigen cross-presentation.

**Figure:**
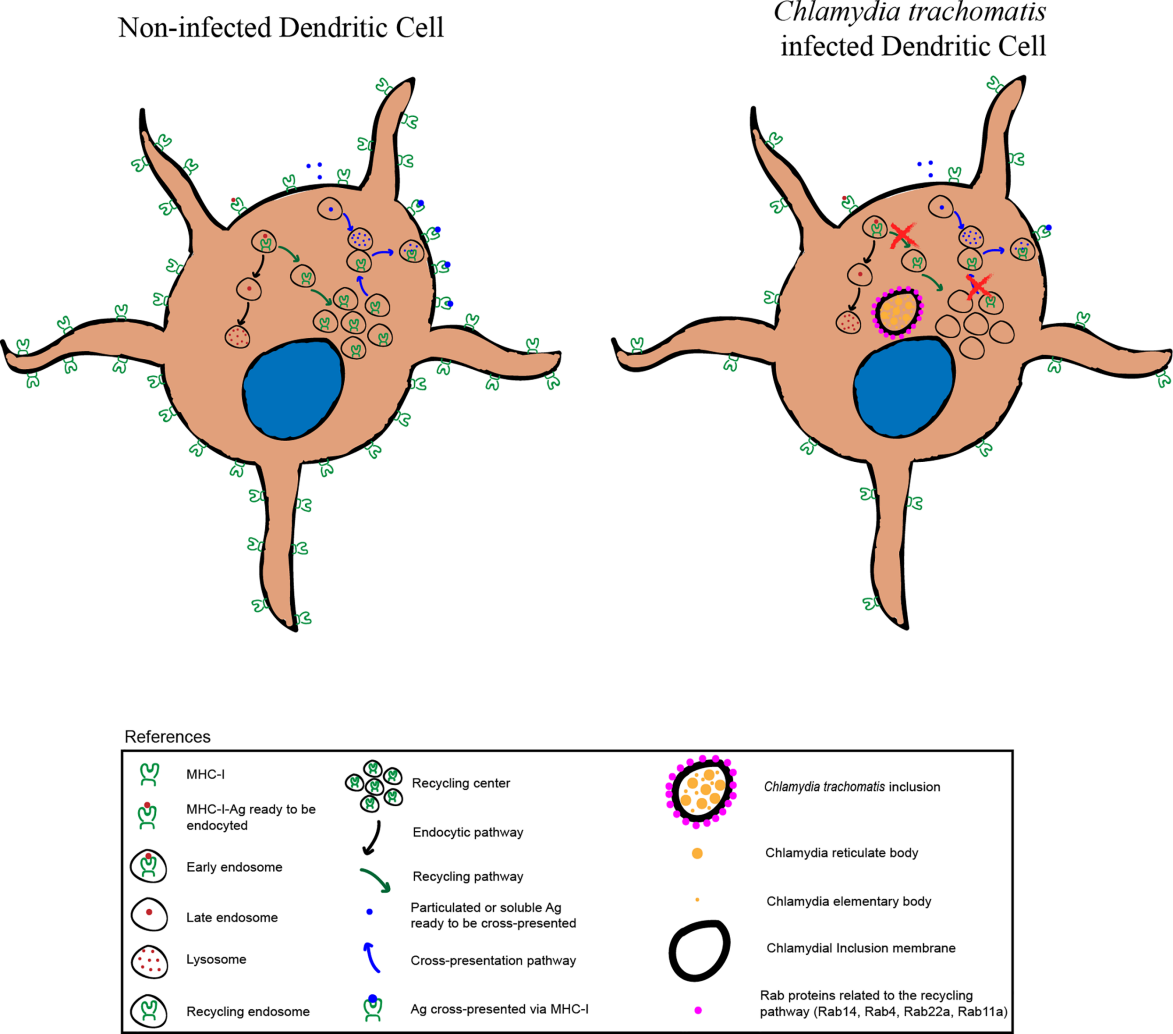
Graphical Abstract

## Introduction

*Chlamydia trachomatis* is an obligate intracellular pathogen and the leading bacterial sexually transmitted infection worldwide. In women, *C. trachomatis* infection is frequently asymptomatic and therefore often remains untreated. This fact may provoke several complications, such as pelvic inflammatory disease, fallopian tube obstruction, ectopic pregnancy, and infertility. In men, this bacterium can cause prostatitis, urethritis, and epididymitis (1, 2). *C. trachomatis* has two morphologically distinct developmental forms: elementary bodies (EBs) and reticulate bodies (RBs). EBs are the infective form that can survive outside the host cell. They adhere to and invade epithelial cells. Once inside, *C. trachomatis* evades the intracellular degradative pathway, shifts to the non-infectious but replicative form known as RB, and converts the phagocytic compartment into a parasitophorous vacuole called inclusion (3–6). For growing and proliferating, *C. trachomatis* acquires essential nutrients from Rab1-, Rab4-, Rab6-, Rab11-, Rab14- and Rab39a-positive vesicles recruited to the inclusion (7–12). After several rounds of replication, RBs differentiate back into EBs, which are released by extrusion or cell lysis, starting the cycle again (13–15). However, when environmental conditions are disadvantageous, *C. trachomatis* enters into a latent, non-replicative form known as the aberrant body (AB), which in turn can revert when the conditions are favorable again. In this sense, *C. trachomatis* can enter and leave the latency state caused by IFN-γ, penicillin treatment, or deprivation of essential nutrients (16).

On one side, *C. trachomatis* has developed multiple strategies to evade the immune system. These include the interference with NF-kB function to avoid the expression of several genes involved in the innate immune response (17, 18), the inhibition of pro-inflammatory cytokines secretion through the stimulation of IL-10 production (19), or the downregulation of MHC-I and -II molecules by the bacterial protease CPAF that induces degradation of the transcription factors RFX-5 and USF-1 (20, 21). These last findings have been questioned and remain controversial (22, 23).

On the other side, *C. trachomatis* infects and develops inside white blood cells, such as macrophages (24, 25) or dendritic cells (DCs) (26–29). DCs are the most potent antigen-presenting cells of the immune system and tightly regulate their endocytic system to process and present antigens efficiently onto MHC molecules (30). This cell type is particularly specialized for achieving cross-presentation, in which exogenous antigen-derived peptides are presented in the context of MHC-I molecules to activate CD8+ T cells (31). Two main pathways have been described for antigen cross-presentation: the vacuolar that is independent of proteasome activity, and the cytosolic, whereby antigens escape from the endo/phagosomal lumen receiving further proteasome-dependent degradation (32). At least four features of DC phagosomes provide these cells with the unique ability to perform efficient antigen cross-presentation: i) a critical regulation of the phagosomal pH (33), ii) an endocytic recycling compartment-dependent MHC-I transport to phagosomes (34, 35), iii) a finely controlled phagosome-to-cytosol export (36) and iv) a special connection between endoplasmic reticulum-derived vesicles and the phagosomal membranes (37, 38) in a mechanism dependent of the SNARE protein Sec22b (39).

In response to chlamydial infection, DCs overexpress B7-1 and ICAM-1; and release IL-1β, IL-6, IL-8, IL-12p70, IL-18, and TNF-α (26), which is at least partially dependent on the engagement of TLR4 (28). However, to date, it has not been described whether *C. trachomatis* perturbs the intracellular transport in DC or if the bacterium impairs any DC function as a consequence of hijacked pathways. Although the ability of *C. trachomatis* infected DCs to initiate normal immune responses has not yet been determined, at least in humans it is clear that a robust immune response is missing. Experimental mice-based setups evinced defects of T cell response, mostly memory CD8+ T cells (40). This phenomenon could explain the typical persistent infection, which leads to a chronic inflammation state with few symptoms, the cause for most harmful health-related difficulties.

In this study, we show that *C. trachomatis* infects JAWS-II cells, a murine DC line, inside which the bacterium intercepts recycling vesicles from the host cell endocytic system. Consequently, chlamydial infection significantly perturbs the transport of MHC-I molecules and reduces the antigen cross-presentation capacity of DCs. Altogether, our data provide new evidence about the hijacking of MHC-I intracellular trafficking in DCs by *C. trachomatis*, supplying relevant information on the hampered ability of the immune system to generate effective CD8+ T cell responses upon chlamydial infection.

## Material And Methods

### Cells and Bacteria

C57BL/6 mice from 6 to 10 weeks of age were used to obtain bone marrow stem cells from the femur and tibia. Animals were maintained in specific pathogen-free conditions, housed in temperature-controlled rooms (22-25°C), and received water and food *ad libitum*. BMDCs were generated after 9 to 14 days of bone marrow cell culture in GM-CSF-containing medium, as previously described (41). BMDCs and the DC line JAWS-II (kindly provided by S. Amigorena) were cultured in Iscove’s Modified Dulbecco’s Medium (IMDM) (GIBCO) with GM-CSF and 10% fetal bovine serum (FBS) (Natocor, Córdoba, Argentina). *C. trachomatis* serovar L2 434/Bu (gently given and typified by Unidad de Estudios de Chlamydias, FFyB, UBA, Bs. As., Argentina) and *C. trachomatis* strain harboring p2TK2-SW2 IncDProm-RSGFP-IncDTerm serovar L2 (GFP) (kindly provided by Isabelle Derré) (42) were used.

For all experiments involving *C. trachomatis* infection, cells and bacteria were centrifuged 20 min at 1200 g and incubated for 24 h or 48 h. For bacterial propagation, HeLa 229 cells (ABAC, Bs.As., Argentina) were infected at a multiplicity of infection (MOI) of 20 and incubated at 37°C in an atmosphere of 5% CO2 and 95% humidified air for 48 h. Then, infected cells were lysed to harvest bacterial particles. Then, EBs were purified as previously described and suspended in 0.2 M sucrose/5% FBS/0.02M phosphate buffer (pH = 7.2). EB concentration was assessed by quantification of Inclusion Forming Units (IFUs) per microliter, as previously described (10).

### Antibodies and Reagents

The following primaries antibodies were used: rabbit anti-CT529 (gently provided by Agathe Subtil, Pasteur Institute, Paris, France), rabbit polyclonal anti-Rab14, Rab4 and Rab11a (Aviva Systems Biology), mouse anti-CD63 (Invitrogen), goat polyclonal anti-EEA1 (Santa Cruz), purified mouse anti-Lamp1, purified mouse anti-H-2K^b^ and FITC-coupled mouse anti-H-2K^b^ (BD Biosciences), mouse monoclonal anti-TfR H68.4 (Invitrogen), mouse monoclonal anti-Rab22a (Santa Cruz), rabbit anti-clathrin (Abcam), rabbit anti-OVA (Sigma-Aldrich), and rabbit anti-MOMP (generously provided by Ted Hackstadt, National Institutes of Health, USA). The secondary antibodies used in this study are goat Cy3-labeled anti-rabbit-IgG, goat Cy3-labeled anti-mouse-IgG, goat anti-mouse-Alexa 647, goat anti-rabbit-Alexa 647 (Molecular Probes), goat horseradish peroxidase (HRP)-conjugated anti-rabbit-IgG and goat HRP-conjugated anti-mouse-IgG antibodies (Jackson ImmunoResearch Laboratories). DNA was stained with DAPI (Life Technologies). Lysotracker Red DND-99 (Life Technologies) was used to stain acidic compartments. The following reagents were also used: ovalbumin (Worthington Biochemical Corporation); 3 µm plain and blue latex beads (Polysciences Inc.); OVA SIINFEKL 257-264 peptide (Polypeptide Group), OVA conjugated to Alexa 488 and DQ-Ovalbumin (Molecular Probes); poly-L-lysine, saponin, protease inhibitor cocktail, BSA (Santa Cruz); Tricine, Tris Base, and TEMED (Calbiochem); Glycine (Bio-Rad); Acrylamide (Promega); Imidazole and NP-40 (ICN Biomedicals Inc.); 7-AAD (Invitrogen). The complete list of antibodies and reagents, with catalog number and references, is provided as **Supplementary Table 1**.

### Immunofluorescence Assays

JAWS-II DCs or BMDCs were placed on poly-L-lysine-coated glass coverslips at room temperature for 30 min. After washing with PBS, a complete medium was added and the cells were incubated for 30 min at 37°C in an atmosphere of 5% CO2. Then cells were infected with *C. trachomatis* L2 or GFP-*C. trachomatis* L2 (MOI 100), centrifuged 20 min at 1200 g and incubated for 24 h. Finally, cells were fixed with 3% PFA for 15 min at room temperature, quenched by adding 0.2 M glycine and washed with PBS. Cells were permeabilized in PBS/0.05% saponin/0.2% BSA for 15 min at room temperature, washed with PBS, and incubated first with primary antibodies overnight at 4°C and then with secondary antibodies for 1 h at room temperature. After washing, the coverslips were mounted with Mowiol supplemented with DAPI. Images were acquired with an Olympus FV-1000 confocal microscope (Olympus, Japan). One z-stack plane is shown from the acquired images. Images were analyzed with the ImageJ software (Fiji).

### Flow Cytometry Analysis

To determine the infection rate of JAWS-II DCs, these cells were infected or not with GFP-*C. trachomatis* L2 at MOI 100, centrifuged 20 min at 1200 g and incubated for 24 h. Cells were fixed with PBS/1% PFA for 10 min on ice, quenched by adding 0.2 M glycine, washed with PBS/1% BSA, and analyzed by FACS.

To study the cell surface expression of MHC-I molecules, JAWS-II DCs non-infected and infected with *C. trachomatis* L2 (24 h pi) were washed with PBS, fixed with 2% PFA on ice during 10 min, and quenched by adding 0.2 M glycine. Then, cells were washed with PBS, incubated with mouse anti-H2Kb (1:200) antibody on ice during 40 min, and washed with PBS/1% BSA. Finally, cells were incubated with Alexa 647-labeled goat anti-mouse (1:200) antibody on ice during 40 min, washed four times with PBS/1% BSA, and analyzed by FACS. Unstained cells were used as a negative control.

To assess fluid-phase endocytosis, JAWS-II DCs non-infected and infected with *C. trachomatis* L2 (24 h pi) were incubated with 0.1 mG/mL or 0.3 mG/mL of OVA soluble coupled to FITC during 1 h at 37°C. And, to evaluate the phagocytic capacity of non-infected and infected JAWS-II DCs with *C. trachomatis* L2 (24 h pi), cells were incubated with 3 µm OVA-coated blue fluorescent beads during 1, 3, and 5 h at 37°C. Incubation with fluorescent soluble and particulate antigens at 4°C was used as negative controls. The endocytic and phagocytic uptake was evaluated by FACS analysis. All these assays were performed by using the FACSAria-III (BD Biosciences) from the Flow Cytometry Facility of the “Facultad de Ciencias Médicas, Universidad Nacional de Cuyo” in Mendoza, Argentina.

### Inclusion Forming Units

Inclusion Forming Units (IFUs) per microliter to quantify EBs harvested from infected DCs were assessed as previously described (10). Briefly, to calculate the amount of EBs generated per infected DC, JAWS-II cells were infected with GFP-*C. trachomatis* L2 at MOI 100, centrifuged 20 min at 1200 g and incubated for 72 h. Then, DCs were incubated for 10 minutes with sterile distilled water and subsequently centrifuged at 4°C for 10 min at 500 rpm to remove cell debris and recover the EBs. SPG buffer (5X) was added to the supernatant until normalized concentration and EBs were purified as previously described (10). Then, serial dilutions of EBs were inoculated onto HeLa cells seeded on 96-well plates. After 24 h, cells were fixed and the inclusions were visualized and counted in 30 fields using a T-2000 Nikon epifluorescence microscope (Nikon, Japan), and expressed as IFUs per µL.

### Immunoblotting

JAWS-II DCs non-infected or infected with *C. trachomatis* L2 (MOI 100) for 24 h were lysed in the presence of protease inhibitor cocktail p-2714 (Sigma-Aldrich, United States) to generate protein extracts. Equal amounts of proteins (30 mg) were resolved in 10% SDS-PAGE gels. Then, proteins were transferred onto 0.45 mm nitrocellulose membranes (Amersham), blocked in PBS/10% milk for 1 h, and subsequently incubated overnight with primary antibodies followed by the corresponding goat anti-rabbit or anti-mouse HRP-conjugated IgG secondary antibody (1:5000). H-2K^b^ total expression was detected with mouse anti-H2-Kb antibody (1:1000). Protein loading was assessed with rabbit anti-clathrin antibody (1:1000) and *C. trachomatis* was detected with rabbit anti-MOMP (1:500). Bound antibodies were revealed using the kit ECL PlusTM (GE Healthcare Life Sciences) in an ImageQuant LAS4000. The intensity of the bands was quantified with the ImageJ software (Fiji) and expressed as arbitrary units.

### MHC-I Recycling Assay

JAWS-II DCs non-infected or infected with GFP-*C. trachomatis* L2 (MOI 100) for 24 h were surface labeled with purified mouse anti-H-2K^b^ antibody followed by an Alexa 647-coupled anti-mouse antibody, both incubated 30 min at 4°C. Then, cells were washed with PBS/1% BSA and incubated for 30 min at 37°C to allow MHC-I internalization. After this incubation, DCs were centrifuged at 1200 rpm for 3 min at 4°C and resuspended in acid buffer stripping solution (0.5 M NaCl and 0.5% acetic acid, pH 3) for 12 min on ice. Then, cells were washed twice with cold PBS and twice with complete medium (pulse), and incubated at 37°C for 0, 10, 20, and 40 min (chase) to allow recycling of MHC-I to the cell surface. After each period, a subset of DCs were fixed as a control of degradation and the others were resuspended again in acid buffer stripping solution for 12 min on ice, washed, and fixed with PBS/1% PFA for 10 minutes on ice to assess MHC-I recycling. All samples were analyzed by FACS. The percentage of recycled or degraded MHC‐I was calculated as described Billadeau DD and collaborators (43), using the equation (T0 – Tx)/T0 × 100. T0 represents the MFI of cells at 0 min, and Tx is the MFI of cells at 10, 20, and 40 min. Differences in the recycling kinetics of non-infected and *C. trachomatis*-infected cells were graphed and analyzed by non-linear regression (P-value = 0.0066).

### Antigen Cross-Presentation Assays

JAWS-II DCs or BMDCs non-infected and infected with *C. trachomatis* L2 (MOI 100) for 12, 24 and 48 h were incubated during 5 h with 3 mg/mL of soluble OVA or 3 µm OVA-coated beads at 37°C, and JAWS-II DCs (non-infected and infected for 24 h) were incubated during 2 h with different concentrations of the SIINFEKL peptide at 37°C. Then, DCs were washed with PBS/0.5% BSA, fixed with 0.008% glutaraldehyde for 3 min at 4°C, quenched with 0.2 M glycine, and washed with PBS. Then, B3Z hybrid T cells were added for 16 h at 37°C. T cell activation was measured by the detection of β-galactosidase activity by optical density (absorbance at 595-655 nm) using CPRG as a substrate for the reaction and expressed as arbitrary units (a.u.). Relative T cell response was the ratio between optical density of the sample and the mean of optical densities of non-infected cells as in Fiegl et al. (44) (**Figures 5A–C** and **Supplementary Figure 7**).

### Antigen Degradation Assays

Non-infected JAWS-II DCs or infected with GFP-*C. trachomatis* L2 (MOI 100) for 24 h were incubated with DQ-OVA (50 µg/mL) for 15 min at 4°C (negative control) or at 37°C (pulse) for probe uptake. After the internalization period, cells were washed three times with cold 2% PBS/BSA and chased at 37°C for 0, 30 and 105 min at 37°C. Then, cells were washed with PBS and fixed with 2% PFA during 10 min at 4°C; and finally, samples were analyzed by FACS. For total intracellular OVA staining, cells incubated for 15 min at 37°C with DQ-OVA (pulse) were fixed and then permeabilized in PBS/0.05% saponin/0.2% BSA for 20 min at room temperature. After that, cells were incubated with rabbit anti-OVA for 2 h at room temperature, then washed three times with 2% PBS/BSA, and subsequently incubated with an anti-rabbit antibody coupled to Alexa 647 for 30 min at 4°C, washed again and analyzed by FACS to assess the total amount of internalized OVA molecules.

### Viability Assay

Non-infected JAWS-II DCs or cells infected with *C. trachomatis* L2 (MOI 100) for 24 h, with or without acid stripping, were labeled with 7-AAD (0.25µg/1.000.000 cells) for 5 min at room temperature. After *in vivo* staining, cells were washed 3 times with PBS, fixed with 2% PFA, and analyzed by FACS. Heat-treated cells (for 10 min at 95°C) were used as a positive control of cell death.

### Statistical Analysis

Student’s unpaired t-test, one-way ANOVA, two-way ANOVA, and the Bonferroni, Sidak or Dunnett post-test were performed as indicated in each experiment. The GraphPad Prism 5 software (CA, USA) was used for all analyses. Data represent mean ± SEM of n experiments. P values less than 0.05 were considered statistically significant.

## Results

### *Chlamydia trachomatis* Infects JAWS-II DCs and Interacts With Recycling Vesicles

**Figure 1:**
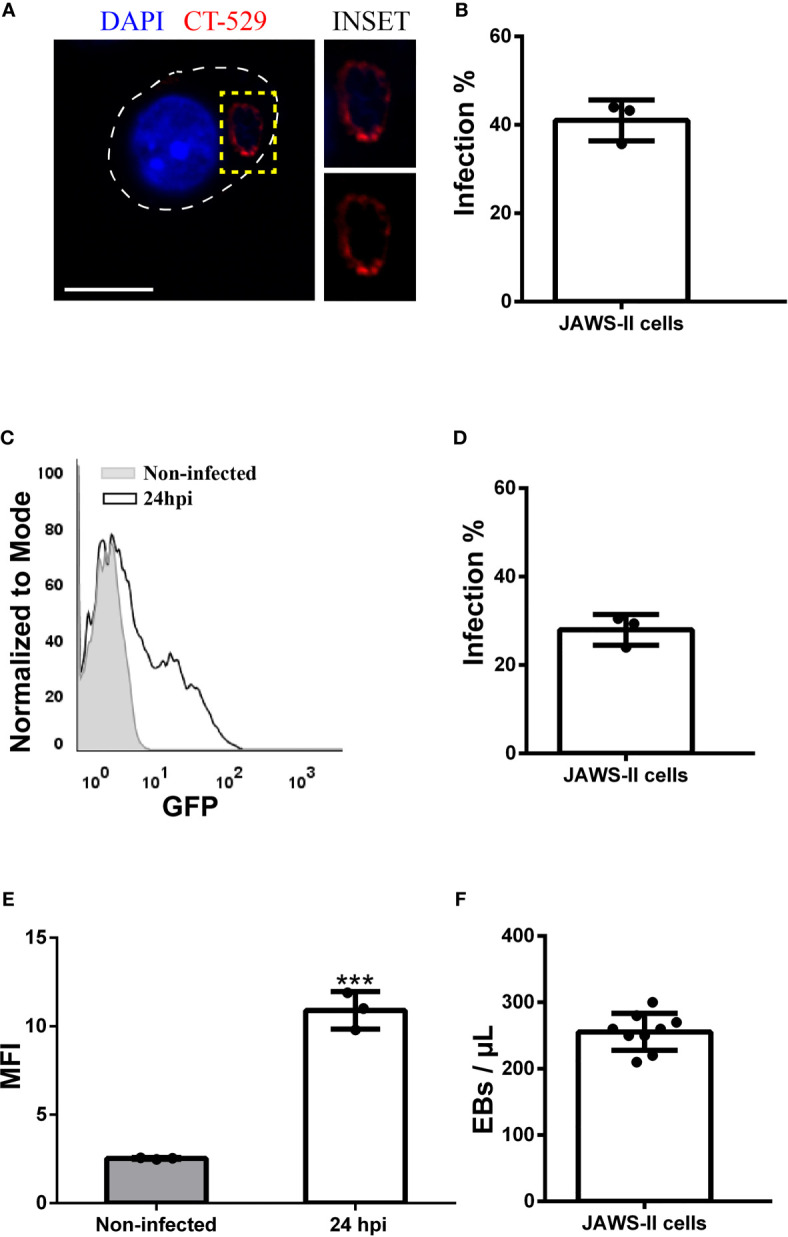
*Chlamydia trachomatis* infects murine JAWS-II DCs. **(A–E)** JAWS-II DCs were infected with *Chlamydia trachomatis* serovar L2 (MOI 100) for 24 and analyzed by **(A, B)** confocal microscopy and **(C–E)** flow cytometry. **(A)** DNA was labeled with DAPI (blue) and the membrane chlamydial inclusion protein CT-529 (red) was detected with a rabbit anti-CT529 antibody and a secondary anti-rabbit Cy3-labeled antibody. Bars represent 10 um. **(B)** Data represent the mean ± SEM (41,00 ± 2,658) of three independent experiments. **(C)** Representative FACS histograms showing the fluorescence intensity of non-infected (grey) and infected (black) JAWS-II DCs. **(D)** Data represent the mean ± SEM (27,97 ± 2,009) of three independent experiments. **(E)** Data show the MFI of GFP and represent the mean ± SEM of three independent experiments. P-value = 0.0002. The two-tailed Student’s unpaired t-test was performed. **(F)** JAWS-II DCs were infected with *C*. *trachomatis* L2 (MOI 100) for 72 h, lysed, and titrated on a monolayer of HeLa cells. Then, the number of EBs produced by infected DCs was calculated. Data represent the mean ± SEM (42,59 ± 1,554) of three independent experiments. ***P < 0.001.

*C. muridarum* (45) and *C. psittaci* (44) infect JAWS-II DCs, but nothing has been reported about the human species *C. trachomatis*. Therefore, we started this study by analyzing if *C. trachomatis* generates productive infections in this murine DC line. First, we infected JAWS-II DCs for 24 h with *C. trachomatis* serovar L2 and analyzed by confocal microscopy the percentage of infected cells after the staining of the chlamydial inclusion membrane protein CT-529 (**Figures 1A, B**). We found that around 40% of JAWS-II DCs were infected and confirmed this observation by flow cytometry analysis. We used a fluorescent strain of *C. trachomatis L2* (GFP-tagged) to infect JAWS-II DCs also for 24 h. With this experimental setup, we evidenced around 30% of infected cells (**Figures 1C, D**), being the mean fluorescent intensity (MFI) of infected JAWS-II DCs significantly higher than non-infected cells (**Figure 1E**). Additionally, we incubated murine BMDCs with GFP-*C. trachomatis* and analyzed the cells by confocal microscopy to demonstrate that these bacteria also infect these cells to a similar extent, developing similar inclusions in shape and perinuclear localization to those generated in JAWs-II DCs (**Supplementary Figure 1**). Besides, we investigated if chlamydial infection induces cell death. For this purpose, we incubated non-infected JAWS-II DCs and cells infected with *C. trachomatis* for 24 h with the dead-cell exclusion marker 7-AAD for 5 min at room temperature before fixation and PerCP fluorescence measurement by flow cytometry. Heat-treated cells were used as a positive control of cell death. As shown in **Supplementary Figure 2**, chlamydial infection does not cause cell death, being the amount of dead cells insignificant in both non-infected and infected cells. Finally, to evaluate EB production, JAWS-II DCs were infected with *C. trachomatis* L2 for 72 h, lysed, and the chlamydial progeny was recovered and used to infect HeLa cells in serial dilutions. As shown in **Figure 1F**, *C. trachomatis* completed its life cycle in JAWS-II DC and generated infective EBs, confirming an effective and productive infection of JAWS-II DCs by this pathogen.

**Figure 2:**
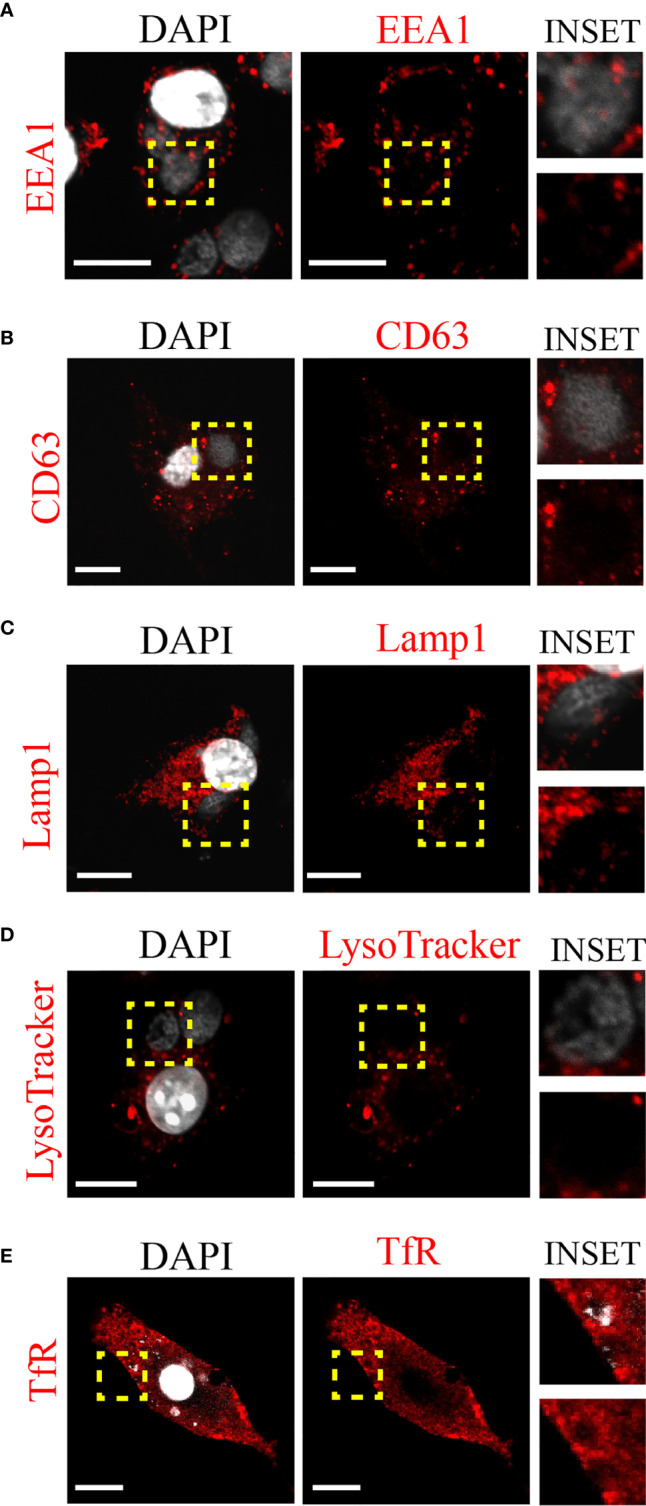
*C. trachomatis* interacts with recycling endosomes in JAWS-II DCs. JAWS-II DCs were infected with *C. trachomatis* L2 (MOI 100) for 24 h and evaluated by confocal microscopy. The intracellular distribution of **(A)** early endosomes (EEA1), **(B)** late endosomes (CD63), **(C)** lysosomes (Lamp1), **(D)** acidic compartments (LysoTracker), and **(E)** recycling endosomes (TfR) were analyzed. Insets show a magnification of the chlamydial inclusion. For each marker, staining with the primary antibody was followed by its corresponding Cy3-conjugated secondary antibody. DAPI was used to stain DNA. Bars represent 10 um. Images are representative of three independent experiments and more than 20 images were analyzed for each endosomal marker.

Next, we decided to investigate whether *C. trachomatis* establishes a close interaction with the DC endocytic network and the nature of this connection. To this aim, we infected JAWS-II DCs with *C. trachomatis* L2 for 24 h and evaluated by immunofluorescence and confocal microscopy the recruitment of different endosomal compartments to the chlamydial inclusion. As shown in **Figure 2A**, the classical early endosomal marker EEA1 did not distribute around the *C. trachomatis* inclusion. Similar results were found with the multivesicular bodies/late endosomal marker CD63 and the late endosome/lysosomal marker Lamp1 (**Figures 2B, C**, respectively). We further confirmed the exclusion of acidic compartments from the bacteria-containing vacuole by labeling infected JAWS-II DCs with the fluorescent dye LysoTracker (**Figure 2D**). Interestingly, when we analyzed the interaction of *C. trachomatis* with transferrin receptor (TfR)-positive recycling vesicles, we observed noticeable recruitment of this marker around the inclusion (**Figure 2E**). These results suggest that *C. trachomatis* establishes very selective interactions with the DC endocytic route and efficiently recruits recycling endosomes.

### Rab Proteins Involved in Endocytic Recycling Are Recruited to the Chlamydial Inclusion

**Figure 3:**
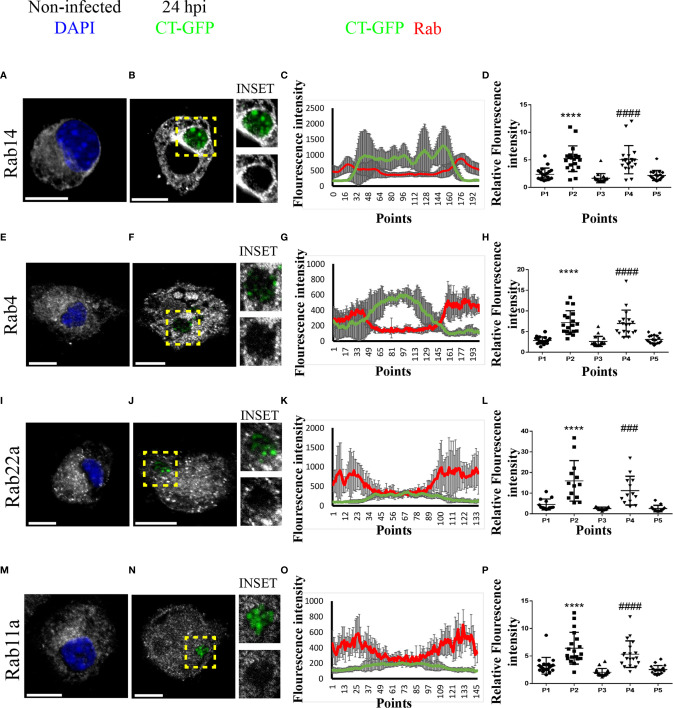
*C. trachomatis* recruits Rab14, Rab4, Rab22a, and Rab11a to the inclusion in JAWS-II DCs. JAWS-II DCs were infected with GFP-*C. trachomatis* L2 at MOI 100 for 24 h and analyzed by confocal microscopy. The intracellular distribution of **(A, B)** Rab14, **(E, F)** Rab4, **(I, J)** Rab22a and **(M, N)** Rab11a was evaluated in non-infected and infected cells. Endogenous Rab proteins were detected by indirect immunofluorescence using primary antibodies followed by its corresponding Cy3-conjugated secondary antibodies. Insets show a chlamydial inclusion magnification for each marker. DAPI was used to stain DNA. Bars represent 10 um. Images are representative of three independent experiments and more than 20 images were analyzed for each Rab. **(C, G, K, O)** A representative chlamydial inclusion was transversely crossed with eight diameter lines to obtain an intensity histogram. Line graphs show the MFI from GFP and the corresponding Rab protein with the SD for each point. **(D, H, L, P)** Chlamydial inclusion was transversely crossed with a diameter line to obtain an intensity histogram for each Rab protein; and five points of each line corresponding to the cytoplasm (P1), inclusion membrane (P2), inside the inclusion (P3), inclusion membrane (P4), and cytoplasm (P5) were selected. Twenty (20) cells were analyzed for each Rab protein. One-way ANOVA with Dunnett’s multiple comparison post-test were performed. ^###^P < 0.001, ^####^P < 0.0001, and ****P < 0.0001.

To characterize the interaction between the chlamydial inclusion and recycling endosomes, we next evaluated the intracellular distribution of different Rab proteins that regulate endocytic recycling transport before and after *C. trachomatis* infection. Thus, we infected JAWS-II DCs with *C. trachomatis*-GFP L2 for 24 h, performed immunofluorescence staining of Rab4, Rab11a, Rab14, and Rab22a, and analyzed the cells by confocal microscopy. Notably, all four Rabs localized around the chlamydial inclusion, to a greater or lesser extent, upon DC infection. The strongest recruitment was observed with Rab14, in which the peripheral distribution of Rab14-positive vesicles (**Figure 3A**) was modified after the infection and accumulated mostly around the chlamydial inclusion (**Figure 3B**). In the case of Rab4 (**Figures 3E, F**), Rab22a (**Figures 3I, J**), and Rab11a (**Figures 3M, N**), the recruitment to the inclusion was weaker than for Rab14, being Rab11a the most modest of the three. Interestingly, the typical perinuclear staining of these Rab proteins corresponding to the recycling center was majorly lost in infected JAWS-II DCs. This observation suggests that *C. trachomatis* may perturb recycling-dependent transport in DCs.

To further analyze the pattern of recruitment of these Rabs around the chlamydial inclusion, we transversely crossed inclusions from B, F, J, and N panels with eight diameter lines in different directions to obtain an intensity histogram for the corresponding GFP-associated Rab protein (**Supplementary Figure 3**). Line graphs obtained showed that Rab14, Rab4, Rab22a, and Rab11a (ordered with respect to their efficiency of recruitment) localized around the chlamydial inclusion following different patterns. According to fluorescence intensity, Rab14 exhibited the greatest and more uniform recruitment to the inclusion (**Figure 3C**). Rab4 also showed a clear recruitment around *C. trachomatis*-containing vacuole, although less uniform than Rab14 (**Figure 3G**). While Rab22a and Rab11a displayed less pronounced and discontinuous recruitment in patches-like patterns, as shown in **Figures 3K, O**, respectively. Additionally, we performed another quantitative analysis whereby each inclusion was transversely crossed with a line, then five points corresponding to the sections cytoplasm (P1), inclusion membrane (P2), inside the inclusion (P3), inclusion membrane (P4), and cytoplasm (P5) were selected to obtain an intensity histogram. For each marker, 20 cells were analyzed, and also with this strategy, all four Rabs exhibited significant increments of intensity at the points corresponding to inclusion boundaries (**Figures 3D, H, L, P**), confirming that recycling Rabs localized around the chlamydial inclusion (**Supplementary Figure 4**).

Altogether, these data support the concept of a relevant role for recycling compartments during DC infection by *C. trachomatis* and suggest that this pathogen could hijack this endocytic pathway, altering important DC functions.

### DC Infection by *Chlamydia trachomatis* Perturbs MHC-I Intracellular Trafficking

**Figure 4:**
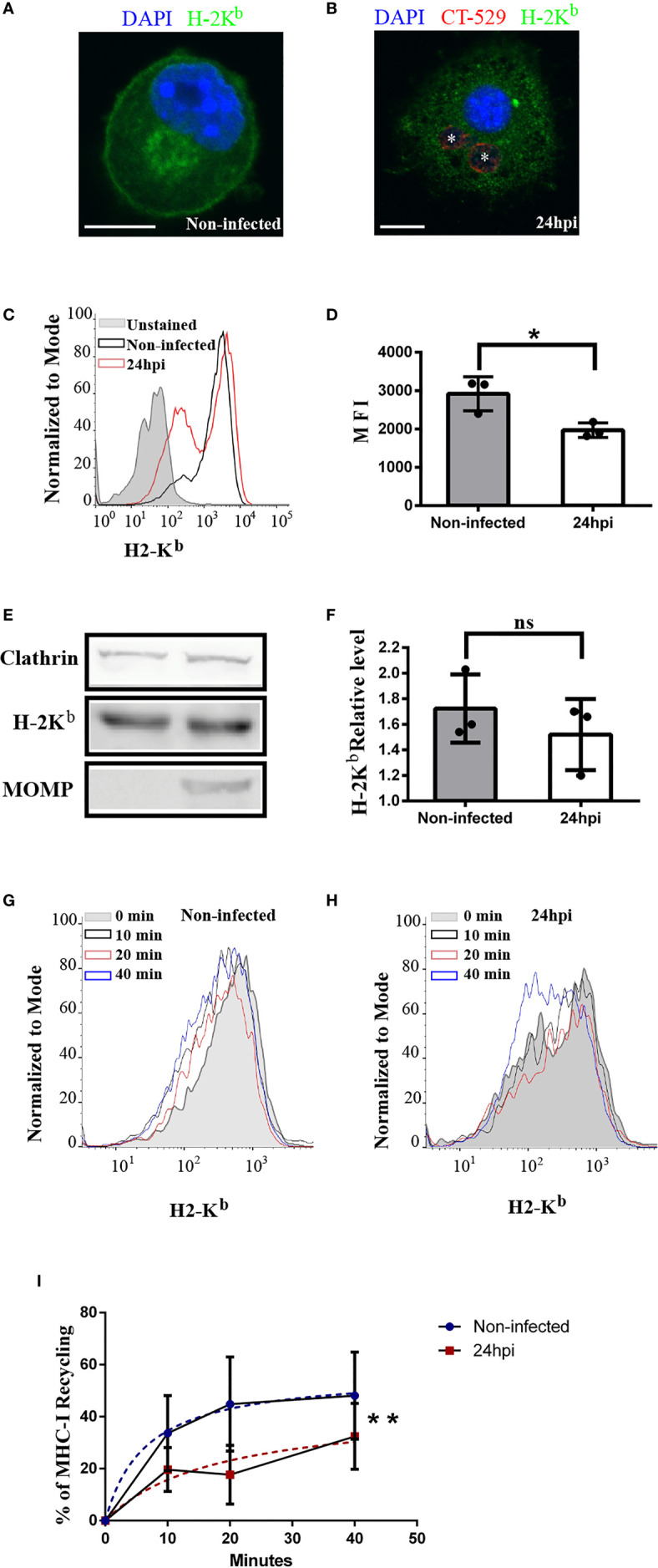
*C. trachomatis* infection alters the intracellular transport of MHC-I molecules in JAWS-II DCs. **(A–F)** JAWS-II DCs were infected with *C. trachomatis* L2 for 24 h at MOI 100 and analyzed by **(A, B)** confocal microscopy, **(C, D)** flow cytometry and **(E, F)** Western blot. **(A, B)** Immunofluorescence staining of MHC-I with an anti-H-2K^b^ mouse monoclonal antibody coupled to FITC showing non-infected **(A)** and infected **(B)** cells. Nuclei were stained with DAPI and chlamydial inclusions were detected with a rabbit anti-CT529 antibody and a secondary anti-rabbit Cy3-labeled antibody. Bars represent 10 um. **(C)** Representative FACS histograms showing the fluorescence intensity of unstained (grey), non-infected (black line), and infected (red line) cells. Cell surface expression of MHC-I molecules was determined with a mouse monoclonal anti-H-2K^b^ antibody and a secondary anti-mouse Alexa 647-conjugated antibody. **(D)** Bar graph shows the MFI of Alexa 647 and represents the mean ± SEM of three independent experiments. P-value = 0.013. The two-tailed Student’s unpaired t-test was performed. **(E)** Immunoblotting of clathrin, H-2K^b^, and MOMP in total cell lysates of non-infected and infected JAWS-II DCs. **(F)** Data represent the densitometry quantification and show the mean ± SEM of three independent experiments. P-value = 0.4127. The two-tailed Student’s unpaired t-test was performed. **(G–I)** MHC-I molecules recycling ability was measured by flow cytometry at the indicated periods by non-infected and infected JAWS-II DCs with *C. trachomatis* L2 for 24 h at MOI 100. **(G, H)** Representative FACS profiles of anti-H-2K^b^ antibody recycling by non-infected **(G)** and infected **(H)** cells. **(I)** The curves show the percentage of anti-H-2K^b^ (Alexa 647) recycling over time and data represent differences between the hyperboles from non-infected (blue line) versus infected (red line) cells of three independent experiments. P-value = 0.0066. No linear regression analysis shows that hyperboles are significantly different. The meaning of the symbol "*" is *P < 0.05 and the P value of NS in **(F)** is 0.4127.

Next, we decided to study the intracellular transport of MHC-I molecules in *C. trachomatis-*infected dendritic cells based on our findings that show the interaction of inclusions with recycling compartments. Then, we infected JAWS-II DCs with *C. trachomatis* L2 for 24 h and we used the anti-mouse H-2K^b^ monoclonal antibody to study the distribution of MHC-I molecules. As shown in **Figures 4A, B** by confocal microscopy, drastic disruption of the perinuclear recycling center containing MHC-I and weaker staining of these molecules at the plasma membrane were evidenced in infected DCs, as compared to non-infected cells. We confirmed and quantified this finding by flow cytometry analysis measuring the MFI of anti-H-2K^b^ staining. MHC-I molecules expression level at the cell surface was significantly reduced in infected JAWS-II DCs (**Figures 4C, D**). Nevertheless, even though MHC-I trafficking to the plasma membrane was affected after *C. trachomatis* infection, the total expression of these molecules was not significantly modified in infected cells, as compared to control non-infected DCs by Western blot analysis (**Figures 4E, F**).

A possible defect of endocytic recycling upon *C. trachomatis* infection could contribute to a defective MHC-I expression at the cell surface. To evaluate this hypothesis, we used a flow cytometry-based approach previously described (35) and we measured MHC-I recycling in JAWS-II DCs. After the steps of binding at 4°C and internalization at 37°C of anti-H-2K^b^, non-infected and infected cells were treated with an acid buffer stripping solution to remove the remaining antibody attached to the plasma membrane. Then, cells were incubated for different periods and the recycled anti-H-2K^b^ was removed from the cell surface through a second acid stripping treatment. Finally, cells were fixed and the percentage of MHC-I recycling was determined as indicated in material and methods. Interestingly, the MHC-I recycling ability of infected JAWS-II DCs was significantly reduced, as compared to non-infected cells (**Figures 4G–I**). This result confirms the significant defect of MHC-I recycling in *C. trachomatis* infected JAWS-II DCs. By the use of the dead-cell exclusion marker 7-AAD, we determined that chlamydial infection and/or acid stripping did not provoke cell death, which was always below the 2% in any experimental condition (**Supplementary Figure 5**).

As control experiments, we assessed the degradation of MHC-I molecules, to evaluate its contribution to the loss of cell associated fluorescence in non-infected and *C. trachomatis* infected JAWS-II DCs. In this subset of cells, after the steps of anti-HG-2Kb binding and internalization, we stripped the cell surface as performed for recycling assays. However, this time we did not perform a second stripping after each chase period to remove the recycled MHC-I molecules from the plasma membrane. In this way, a decrease in the cell associated fluorescence intensity was only due to the intracellular degradation of MHC-I molecules. Our results show that after 20 min of chase, MHC-I degradation was not significant and only at 40 min there was a mild degradation of these molecules, mainly in non-infected cells (**Supplementary Figure 6**).

Altogether, these data indicate that the MHC-I intracellular transport is significantly compromised in DCs when these cells are infected by the bacterium *C. trachomatis*, which could result in disadvantageous implications for the immune system.

### *Chlamydia trachomatis* Infection Hampers the Antigen Cross-Presentation Ability of DCs

**Figure 5:**
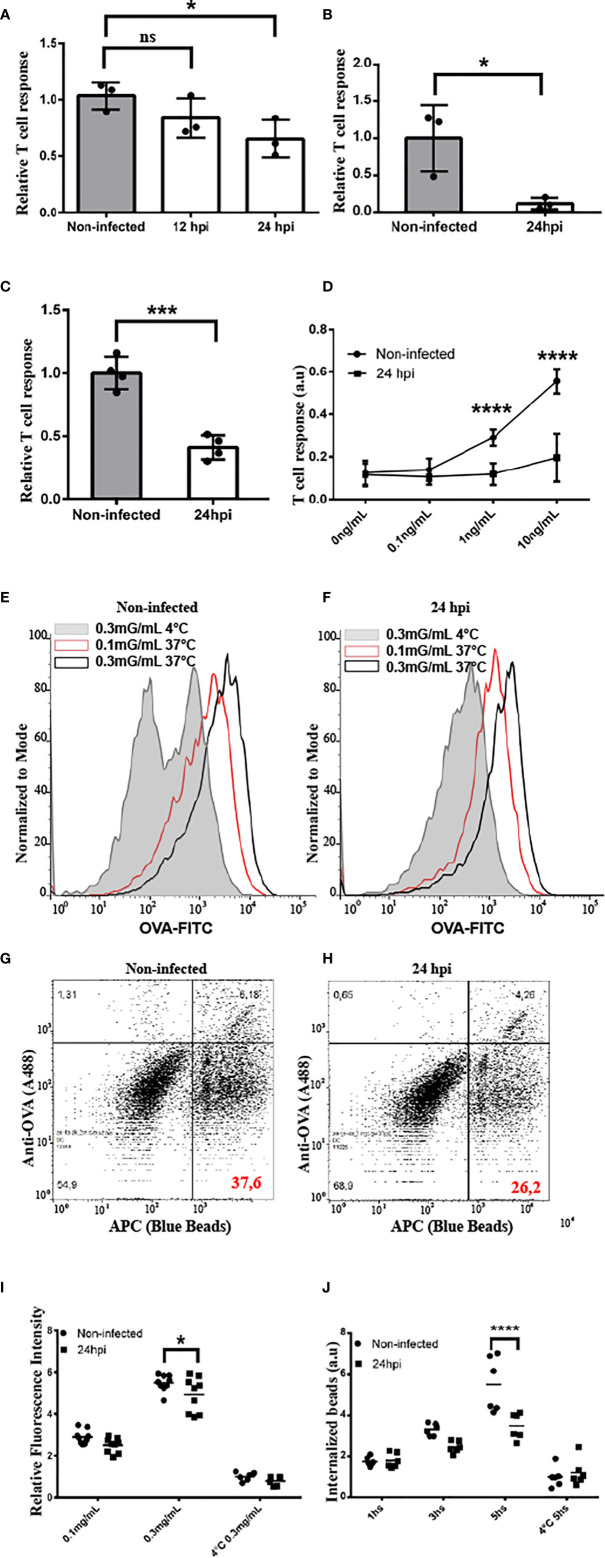
*C. trachomatis* disturbs antigen cross-presentation by JAWS-II DCs. **(A–D)** The cross-presentation ability of non-infected and infected JAWS-II DCs with *C*. *trachomatis* L2 at MOI 100 after incubation with **(A)** soluble OVA, **(B)** OVA/BSA-coated beads, **(C)** soluble OVA (BMDCs), or **(D)** the SIINFEKL control peptide at the indicated concentrations was evaluated with the B3Z T cell hybridoma. In **(A, D)** one-way ANOVA and Bonferroni’s multiple comparisons tests were performed. *P < 0.05 and ****P < 0.0001. In **(B, C)** two-tailed Student’s unpaired t-tests were performed *P = 0.0280 (for **B**) and ***P = 0.0003 (for **C**). Data represent mean ± SEM of three (for **A–C**) and four (for **D**) independent experiments. **(E, F)** Non-infected and infected JAWS-II DCs with *C. trachomatis* L2 for 24 h at MOI 100 were incubated for 1 h with the indicated concentrations of OVA coupled to FITC and fluid-phase endocytosis was assessed by flow cytometry. Effective uptake was performed at 37°C and the highest concentration of OVA at 4°C was used as control. Representative FACS histograms showing the fluorescence intensity of FITC at the different conditions used in non-infected **(E)** and infected **(F)** cells. **(G, H)** Non-infected and infected JAWS-II DCs with *C. trachomatis* L2 for 24 h at MOI 100 were incubated during 1, 3, and 5 h with 3 μm fluorescent latex beads, and the phagocytic capacity was assessed by flow cytometry. Phagocytosis was conducted at 37°C for effective uptake and at 4°C for 5 h as control. Representative FACS histograms of the condition 5 h at 37°C showing the APC+/OVA- regions indicate the percentage of internalized particles. **(I)** Data represent the endocytic uptake and the mean ± SEM of three independent experiments (n = 6). *P < 0.05. Two-way ANOVA and Sidak’s multiple comparisons tests were performed. **(J)** Data represent phagocytosis and the mean ± SEM of three independent experiments (n = 6). ****P < 0.0001. Two-way ANOVA and Sidak’s multiple comparisons tests were performed. P-value of NS in **(A)** is P > 0.05.

Because MHC-I recycling is an important feature of antigen cross-presentation, we aimed to examine if this immune process is jeopardized by chlamydial infections. First, we infected JAWS-II DCs with *C. trachomatis* L2 for 12 or 24 h and incubated them with the soluble model antigen ovalbumin (OVA). As shown in **Figure 5A**, antigen cross-presentation was partially inhibited upon DC infection and this defect was more important at 24 than 12 h post-infection. Also, the cross-presentation of 3 µm OVA-coated latex beads was significantly reduced by infected DCs, as compared to non-infected cells (**Figure 5B**). Interestingly, similar results were obtained when primary bone marrow-derived DCs (BMDCs) were infected during 24 h and soluble OVA cross-presentation was evaluated. Also in this experimental setup, the antigen cross-presentation ability of infected DCs was significantly affected, as compared to non-infected cells (**Figure 5C**). In agreement with the finding of less MHC-I cell surface expression in infected cells (**Figures 4C, D**), the presentation of the SIINFEKL peptide, which does not need further intracellular processing, was also significantly reduced in infected JAWS-II DCs (**Figure 5D**). Interestingly, cross-presentation of soluble and particulate antigens by *C. trachomatis*-infected DCs remains significantly hampered after 48 h post-infection (**Supplementary Figure 7**).

Finally, we decided to test whether the chlamydial infection has an impact on antigen uptake by DCs. Thus, we infected JAWS-II DCs with *C. trachomatis* L2 for 24 h and incubated them either with fluorescent soluble OVA or 3 µm OVA-coated fluorescent beads to evaluate fluid-phase endocytosis and phagocytosis, respectively. As shown in **Figures 5E, F, I**, after 1 h of soluble OVA continuous uptake, the amount of antigen inside infected DCs is slightly diminished, as compared to non-infected cells, and a significant difference is evidenced only with the highest concentration of OVA incubated at 37°C. Similarly, when we analyzed internalized fluorescent particles, which are negative for anti-OVA staining (**Figures 5G, H**), we observed a reduction of the phagocytic capacity in infected DCs only after 5 h of internalization, but not at earlier time points or for the binding at 4°C (**Figure 5J**). In line with these experiments, we used other approach to investigate if chlamydial infection modifies cellular ability to degrade antigens. Briefly, non-infected and *C. trachomatis* infected JAWS-II DCs were incubated with DQ-OVA for 15 min at 37°C (internalization period) followed by 0, 30 and 105 min of chase at 37°C. DQ-OVA develops green fluorescence upon degradation. Our results show that *C. trachomatis* infected DCs degraded this soluble antigen in a similar extent to non-infected cells. We also assessed total intracellular OVA (non-fluorescent and fluorescent DQ-OVA molecules) by staining with an anti-OVA antibody coupled to Alexa 647. Results indicated that *C. trachomatis* infected JAWS-II DCs display a slight reduction in the amount of internalized OVA while they exhibit a similar antigen degradation capacity compared to non-infected cells (**Supplementary Figure 8**).

Overall, our results provide compelling evidence about the significant changes that *C. trachomatis* causes on DC functions, hijacking the host cell endocytic route through the recruitment of recycling vesicles. This interaction between the chlamydial inclusion and recycling endosomes directly impacts the proper MHC-I intracellular trafficking and cross-presentation ability of infected DCs.

## Discussion

*C. trachomatis*, as most intracellular pathogens, constitutes a challenge to the immune system. Once internalized, the bacterium actively modified the phagosomal membrane to generate a protective vacuole, the inclusion that diverts from the phagocytic pathway and avoids lysosomal degradation. One effective strategy developed by *C. trachomatis* to subvert intracellular trafficking is the hijacking of Rab GTPases (3, 46). Our findings show that in DCs, *C. trachomatis* recruits Rab4, Rab11a, Rab14, and Rab22a to the inclusion boundaries. These GTPases control different steps of the recycling transport (47). Beyond differences in the pattern or intensity of Rab associated with the inclusion, these proteins confer the bacterium ability to interact with the endocytic recycling compartment. This compartment emerges as an important hub during the intracellular transport of exogenous antigens in DCs, particularly during MHC-I cross-presentation. Furthermore, Rab11a and Rab22a were shown to localize to MHC-I in the endosomal recycling compartment and both are necessary for efficient cross-presentation (34, 35). Rab14 and the insulin-responsive aminopeptidase (IRAP) associate with antigen-containing phagosomes in CD8+ DCs and promote the MHC-I cross-presentation pathway (48). Given that a fundamental issue for antigen cross-presentation is MHC-I recycling to the cell surface, *C. trachomatis* could interfere with the transport of these molecules to hide from the immune system and persist intracellularly. Precisely, characteristic features of chlamydial infections are persistence and chronicity, bases of the pathological damage to the female reproductive system (49).

Chlamydial protease-like activity factor (CPAF), tail-specific protease (Tsp), and chlamydial high temperature requirement protein A (cHtrA) hinder cross-presentation by selective degradation of bacterial T cell antigens (50), downregulation of MHC-I molecules at the cell surface (20, 51), and cleavage of transcription factors RFX-5, NF-κB p65, and USF-1 (21, 23, 52). In our experiments, *C. trachomatis* infection dramatically changes MHC-I subcellular distribution without affecting its total intracellular expression. Confocal microscopy and flow cytometry approaches show the loss of MHC-I molecules at the cell surface and the perinuclear recycling center after infection. In agreement with our results, Kägebein et al. reported that MHC-I expression remains unaltered after chlamydial infection in epithelial cells and fibroblasts; however, MHC-I exposition at the cell surface does not vary in these cells (23). Given that MHC-I recycling is essential for exogenous antigens presentation to CD8+ T cells, by analyzing the OVA-based model, we found that cross-presentation of both, soluble and particulate antigens, is impaired upon *C. trachomatis* infection of murine JAWS-II DCs or primary BMDCs. Besides, the presentation of the peptide SIINFEKL that is independent of intracellular processing and the endo/phagocytic capacity of DCs also decreases after infection.

Our findings support the notion that by interfering with Rab-controlled intracellular transport, *C. trachomatis* hijacks antigen cross-presentation and conspires against an efficient cytotoxic CD8+ T cell immune response, turning DCs into a convenient vehicle for bacterial spreading. In mice, *C. muridarum* spreads from genital tissues to the gastrointestinal tract in a staged transport involving genital tissue/ganglion, ganglion/spleen, and spleen/gastrointestinal tract (53, 54). Accordingly, in humans *C. trachomatis* was found in the intestine regardless of sexual behavior, suggesting a putative similar spreading way (54–56). Furthermore, the human respiratory pathogen *C. pneumoniae* disseminates from the lungs to the body through infected monocyte-derived DCs (57).

Several chlamydial proteins are presented by MHC-I on infected cells, such as CT442 and CT529, becoming good candidates for vaccine development. Despite recombinant virus expressing CT442 triggers robust specific CD8+ T cell response, more than the bacterium itself, it fails to eliminate *C. trachomatis* from the infected tissues and does not confer protection against a subsequent challenge (58–61). Our data show that the chlamydial infection disturbs DCs functioning; therefore, it could produce a defective CD8+ T cell immune response against *C. trachomatis*, and possibly also against other pathogens. Starnbach and colleagues demonstrated that *C. trachomatis* infection fails to develop memory CD8+ T cells, providing insufficient protection to later chlamydial exposition that explains the high rate of re-infection. Besides, mice co-infected with *Chlamydia* and *Lysteria monocytogenes* display a dose-dependent decrease in *Listeria*-specific immune response, indicating that *Chlamydia* impairs the priming of heterologous CD8+ T cells (62). Our study provides new evidence that chlamydial infection distorts intracellular trafficking in DCs, reducing MHC-I recycling transport, thus, restricting pathogen-derived antigens become available for presentation to T lymphocytes. The interference with priming/activation of naive CD8+ T cells circumvents the cross-presentation pathway and avoids infected cell recognition by cytotoxic T cells. Ultimately, hiding antigens intracellularly favors pathogens to grow unhindered within the infected cell. Our data shed light on a new mechanism that likely contributes to the failure in generating a protective CD8+ T cell response. Altogether, our findings explain, in part, actual difficulties in vaccine development and the high rate of persistent chlamydial infections and co-infections with other intracellular pathogens, such as HIV (63).

Cross-presentation signals the presence of intracellular pathogens to cytotoxic T lymphocytes, which eliminate infected cells by the recognition of pathogen-derived peptides loaded onto MHC-I molecules at the cell surface. Unraveling how intracellular pathogens interfere with MHC-I antigen presentation to avoid clearance by the immune system will pave the way to a rational design of protective vaccines to halt the worldwide spreading of these infections.

## Data Availability Statement

The raw data supporting the conclusions of this article will be made available by the authors, without undue reservation.

## Ethics Statement

The animal study was reviewed and approved by Comité Institucional para el Cuidado y Uso de Animales de Laboratorio (CICUAL), Facultad de Ciencias Médicas, Universidad Nacional de Cuyo.

## Author Contributions

DD and AC performed all experiments and prepared the figures. IC and MD conceived, designed, and supervised the project. All authors discussed results, data analysis, commented on the manuscript preparation and wrote the manuscript. All authors contributed to the article and approved the submitted version.

## Funding

This work was supported by the “Agencia Nacional de Promoción Científica y Tecnológica” (PICT 2016-0013 to IC, PICT 2016-1851 and 2018-03737 to MD). We acknowledge the financial support by the “Consejo Nacional de Investigaciones Científicas y Técnicas” (CONICET) from Argentina.

## Conflict of Interest

The authors declare that the research was conducted in the absence of any commercial or financial relationships that could be construed as a potential conflict of interest.

## Supplementary Material

Supplementary Table 1 List of antibodies and reagents

Supplementary Figure 1 C*. trachomatis* can infect murine BMDCs. BMDCs were infected with GFP-*C. trachomatis* L2 (MOI 100) for 24 h and analyzed by confocal microscopy. DNA was labeled with DAPI (blue). Insets show a magnification of the chlamydial inclusion. Bar represents 10 µm.

Supplementary Figure 2 C*. trachomatis* infection does not affect JAWS II cells viability. Non-infected JAWS II DCs and cells infected with C. trachomatis for 24 h (24 hpi) were incubated with the cell death marker 7-AAD viability marker and analyzed by flow cytometry. Heated-treated cells were used as a control of dead cells. **(A)** Representative FACS profiles show MFI (PerCP) corresponding to the presence of dead cells in the sample. **(B)** Graph bar represents the mean percentage of dead cells in each condition.

Supplementary Figure 3 Rab proteins involved in endocytic recycling are recruited to the chlamydial inclusion. JAWS-II DCs were infected with GFP-*C. trachomatis* L2 at MOI 100 for 24 h and analyzed by confocal microscopy. Endogenous Rab14, Rab4, Rab22a and Rab11a proteins were detected by indirect immunofluorescence using primary antibodies followed by its corresponding Cy3-conjugated secondary antibodies. Images are representative of three independent experiments. Each chlamydial inclusion was transversely crossed by eight diameter lines to obtain an intensity histogram. Line graphs show the intensity histogram of each Rab. More than 20 images were analyzed for each Rab using the ImageJ software (Fiji).

Supplementary Figure 4 Statistical analysis of Rab14, Rab4, Rab22a and Rab11a fluorescence intensity in *C. trachomatis*-infected DCs. The table displays the statistical significance of Rab14, Rab4, Rab22a and Rab11a fluorescence intensity comparing cytoplasm (P1), inclusion membrane (P2), inside the inclusion (P3), inclusion membrane (P4), and cytoplasm (P5) corresponding to panels 3D, 3H, 3L and 3P. One-way ANOVA and Dunnett’s multiple comparison post-test were performed. ^#^P < 0.05, ^##^P < 0.01, ^###^P < 0.001, ^####^P < 0.0001, and ****P < 0.0001.

Supplementary Figure 5 Chlamydial infection and acid stripping does not affect cell viability. **(A–D)** JAWS II cells viability was measured by flow cytometry using 7-AAD viability marker. A) Representative FACS histograms show 7-AAD MFI corresponding to unstained (grey), non-infected (red line) and *C. trachomatis* infected cells (24 hpi) (black line). Cells death by heating was used as a positive control (green line). **(B**, **D)** Graph bar represents the mean percentage of dead cells in each experimental condition. **(C)** Representative FACS histograms show 7-AAD MFI of non-infected cells (NI, black line), non-infected cells treated with stripping buffer (NI + Stripping, blue line), and cells infected with *C. trachomatis* for 24h and treated with stripping buffer (24hpi + Stripping). Cells death by heating was used as a positive control (green line).

Supplementary Figure 6
*C. trachomatis* does not alter MHC-I degradation. MHC-I degradation was measured by flow cytometry at the indicated time points in non-infected JAWS-II DCs and cells infected with *Chlamydia trachomatis* L2 for 24 h at MOI 100. Representative FACS profiles of anti-H-2Kb antibody degradation by non-infected **(A)** and infected **(B)** cells. **(C)** The curves show the percentage of anti-H-2Kb (Alexa 647) degraded over time.

Supplementary Figure 7 C*. trachomatis* alters the antigen cross-presentation ability of DCs at 48 h post-infection. The cross-presentation ability of non-infected and infected JAWS-II DCs with *C. trachomatis* L2 for 48 h at MOI 100 after incubation with **(A)** soluble OVA, **(B)** OVA/BSA-coated beads, **(C)** soluble OVA (BMDCs) was evaluated with the B3Z T cell hybridoma. Two-tailed Student’s unpaired t-tests were performed. *P < 0.0265, ***P < 0.0003, and ****P < 0.0001.

Supplementary Figure 8 Chlamydial infection does not affect soluble antigen degradation. **(A-C)** Representative FACS profiles shows the MFI corresponding to DQ-OVA degradation in non-infected JAWS-II DCs **(A)** and cells infected with *C. trachomatis* L2 for 24 h (MOI 100) **(B)**. Cells were incubated for 15 min at 4°C (negative control) or 15 min at 37°C (pulse) with DQ-OVA. Then, cells were incubated for 0, 30 and 105 min at 37°C (chase period). **(C)** Quantification of the soluble DQ-OVA degradation measured by flow cytometry at the indicated time periods. Data represent FITC MFI. **(D)** Representative FACS profiles show the MFI corresponding to the total amount of soluble DQ-OVA internalized in non-infected and infected JAWS-II DCs during the pulse period (15 min at 37°C). Cells were fixed, permeabilized and labeled with an anti-OVA antibody followed by a secondary antibody conjugated with Alexa 647. **(E)** Quantification of total OVA staining (assessed by Alexa 647 MFI) after DQ-OVA internalization in non-infected and infected JAWS-II DCs.
