# Supplementary figures and images for: Chlamydia trachomatis Infection Impairs MHC-I Intracellular Trafficking and Antigen Cross-Presentation by Dendritic Cells
Source: Front Immunol. 2021 Apr 15;12:662096. doi: 10.3389/fimmu.2021.662096 (PMC8082151; doi:10.3389/fimmu.2021.662096)

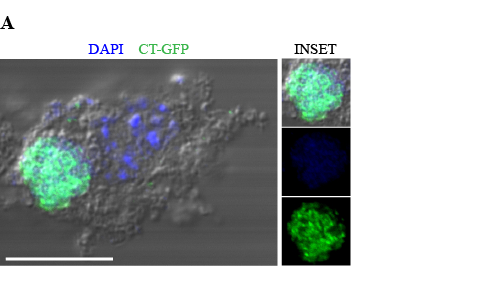

Supplement: Supplementary Figure 1 — C. trachomatis can infect murine BMDCs. BMDCs were infected with GFP-C. trachomatis L2 (MOI 100) for 24 h and analyzed by confocal microscopy. DNA was labeled with DAPI (blue). Insets show a magnification of the chlamydial inclusion. Bar represents 10 µm. [file Image_1.tif]

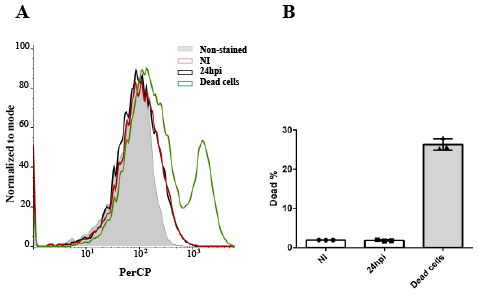

Supplement: Supplementary Figure 2 — C. trachomatis infection does not affect JAWS II cells viability. Non-infected JAWS II DCs and cells infected with C. trachomatis for 24 h (24 hpi) were incubated with the cell death marker 7-AAD viability marker and analyzed by flow cytometry. Heated-treated cells were used as a control of dead cells. (A) Representative FACS profiles show MFI (PerCP) corresponding to the presence of dead cells in the sample. (B) Graph bar represents the mean percentage of dead cells in each condition. [file Image_2.tif]

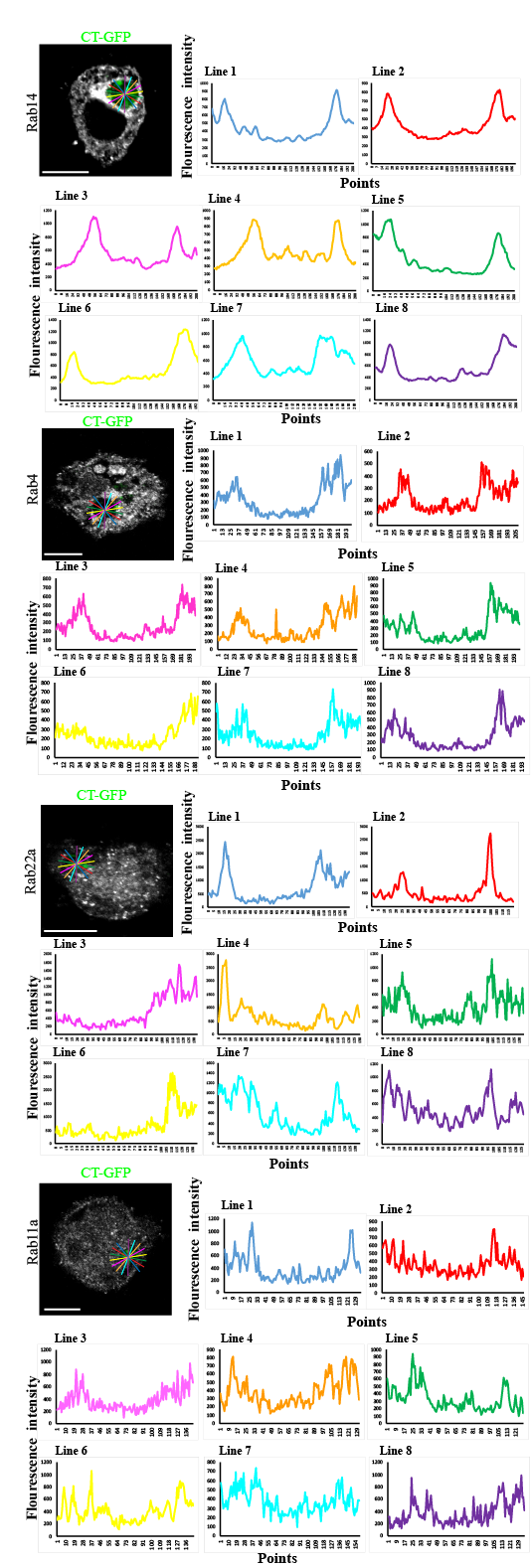

Supplement: Supplementary Figure 3 — Rab proteins involved in endocytic recycling are recruited to the chlamydial inclusion. JAWS-II DCs were infected with GFP-C. trachomatis L2 at MOI 100 for 24 h and analyzed by confocal microscopy. Endogenous Rab14, Rab4, Rab22a and Rab11a proteins were detected by indirect immunofluorescence using primary antibodies followed by its corresponding Cy3-conjugated secondary antibodies. Images are representative of three independent experiments. Each chlamydial inclusion was transversely crossed by eight diameter lines to obtain an intensity histogram. Line graphs show the intensity histogram of each Rab. More than 20 images were analyzed for each Rab using the ImageJ software (Fiji). [file Image_3.tif]

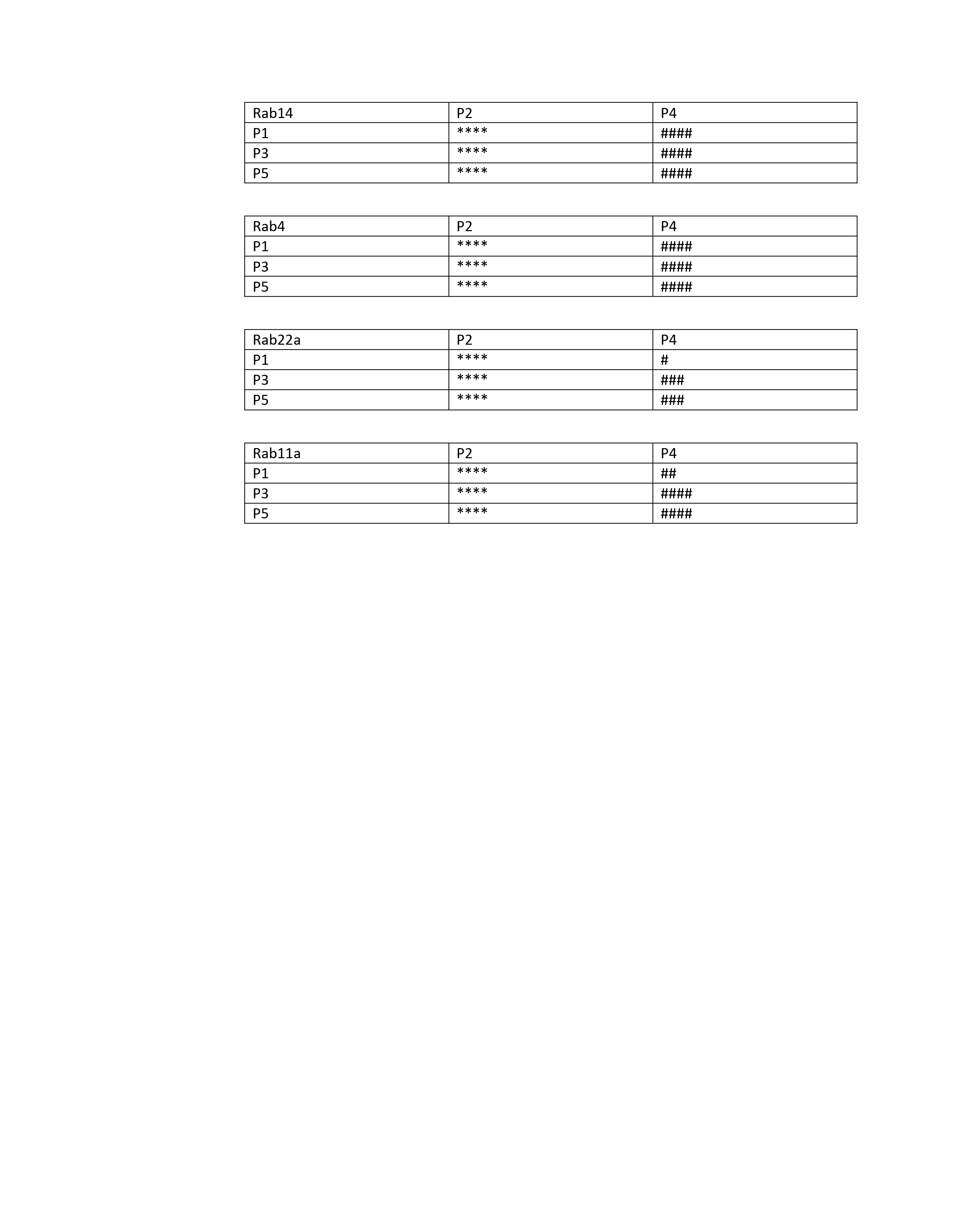

Supplement: Supplementary Figure 4 — Statistical analysis of Rab14, Rab4, Rab22a and Rab11a fluorescence intensity in C. trachomatis-infected DCs. The table displays the statistical significance of Rab14, Rab4, Rab22a and Rab11a fluorescence intensity comparing cytoplasm (P1), inclusion membrane (P2), inside the inclusion (P3), inclusion membrane (P4), and cytoplasm (P5) corresponding to panels 3D, 3H, 3L and 3P. One-way ANOVA and Dunnett’s multiple comparison post-test were performed. #P < 0.05, ##P < 0.01, ###P < 0.001, ####P < 0.0001, and ****P < 0.0001. [file Image_4.tif]

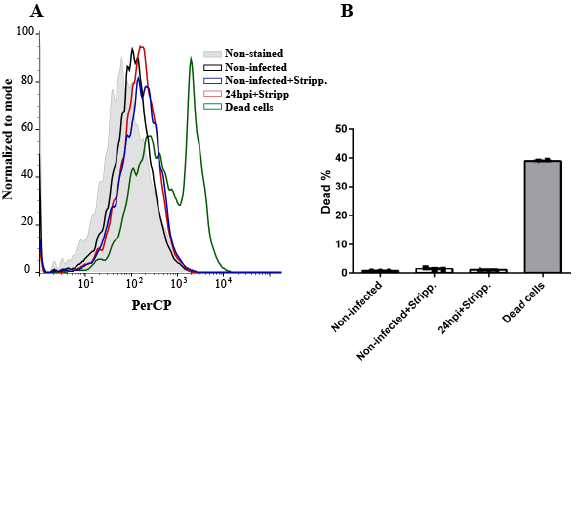

Supplement: Supplementary Figure 5 — Chlamydial infection and acid stripping does not affect cell viability. (A–D) JAWS II cells viability was measured by flow cytometry using 7-AAD viability marker. A) Representative FACS histograms show 7-AAD MFI corresponding to unstained (grey), non-infected (red line) and C. trachomatis infected cells (24 hpi) (black line). Cells death by heating was used as a positive control (green line). (B, D) Graph bar represents the mean percentage of dead cells in each experimental condition. (C) Representative FACS histograms show 7-AAD MFI of non-infected cells (NI, black line), non-infected cells treated with stripping buffer (NI + Stripping, blue line), and cells infected with C. trachomatis for 24h and treated with stripping buffer (24hpi + Stripping). Cells death by heating was used as a positive control (green line). [file Image_5.tif]

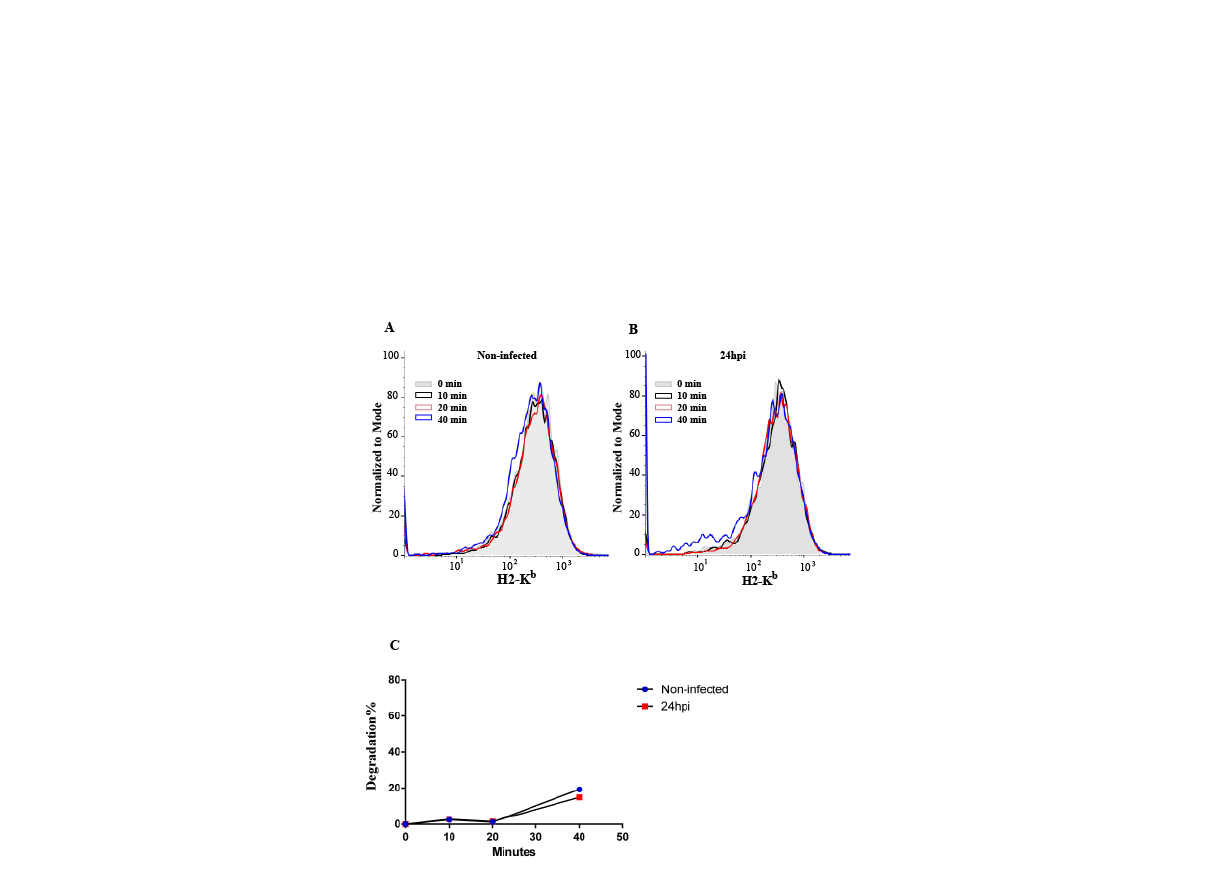

Supplement: Supplementary Figure 6 — C. trachomatis does not alter MHC-I degradation. MHC-I degradation was measured by flow cytometry at the indicated time points in non-infected JAWS-II DCs and cells infected with Chlamydia trachomatis L2 for 24 h at MOI 100. Representative FACS profiles of anti-H-2Kb antibody degradation by non-infected (A) and infected (B) cells. (C) The curves show the percentage of anti-H-2Kb (Alexa 647) degraded over time. [file Image_6.tif]

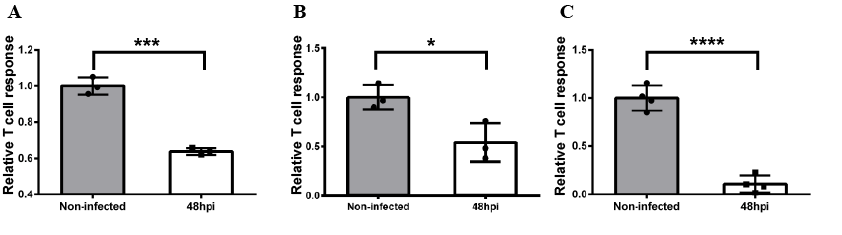

Supplement: Supplementary Figure 7 — C. trachomatis alters the antigen cross-presentation ability of DCs at 48 h post-infection. The cross-presentation ability of non-infected and infected JAWS-II DCs with C. trachomatis L2 for 48 h at MOI 100 after incubation with (A) soluble OVA, (B) OVA/BSA-coated beads, (C) soluble OVA (BMDCs) was evaluated with the B3Z T cell hybridoma. Two-tailed Student’s unpaired t-tests were performed. *P < 0.0265, ***P < 0.0003, and ****P < 0.0001. [file Image_7.tif]

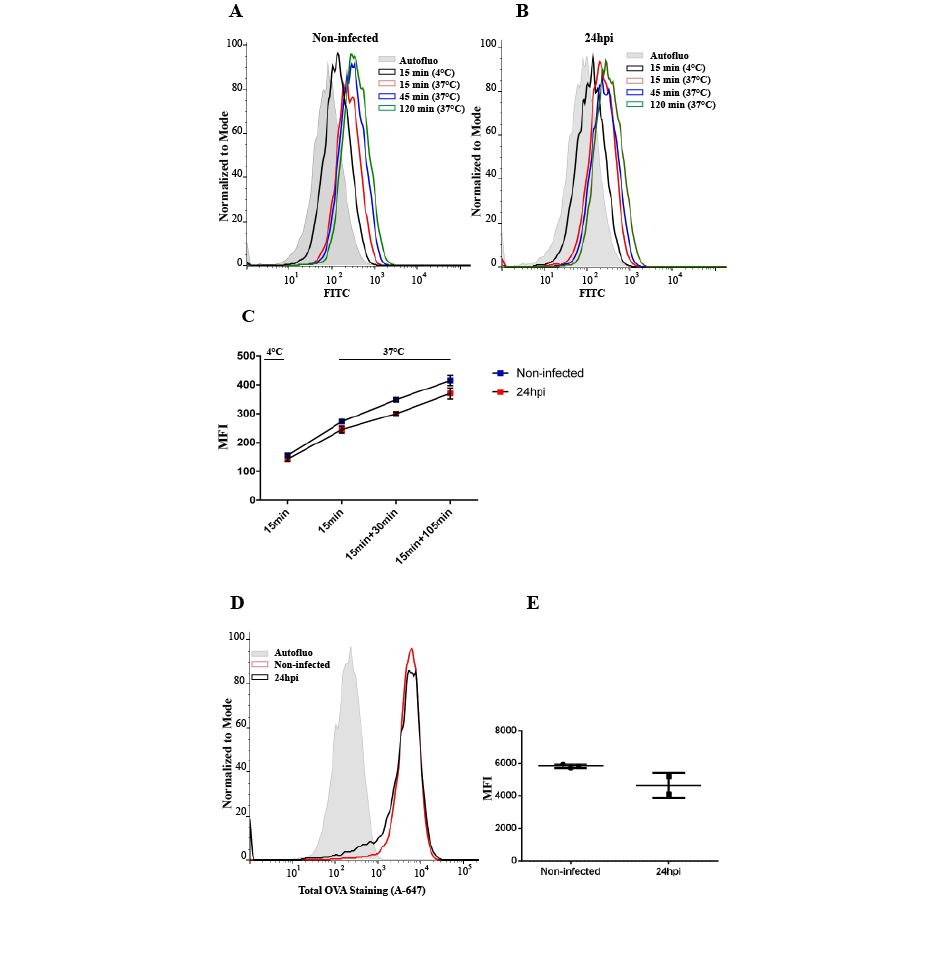

Supplement: Supplementary Figure 8 — Chlamydial infection does not affect soluble antigen degradation. (A-C) Representative FACS profiles shows the MFI corresponding to DQ-OVA degradation in non-infected JAWS-II DCs (A) and cells infected with C. trachomatis L2 for 24 h (MOI 100) (B). Cells were incubated for 15 min at 4°C (negative control) or 15 min at 37°C (pulse) with DQ-OVA. Then, cells were incubated for 0, 30 and 105 min at 37°C (chase period). (C) Quantification of the soluble DQ-OVA degradation measured by flow cytometry at the indicated time periods. Data represent FITC MFI. (D) Representative FACS profiles show the MFI corresponding to the total amount of soluble DQ-OVA internalized in non-infected and infected JAWS-II DCs during the pulse period (15 min at 37°C). Cells were fixed, permeabilized and labeled with an anti-OVA antibody followed by a secondary antibody conjugated with Alexa 647. (E) Quantification of total OVA staining (assessed by Alexa 647 MFI) after DQ-OVA internalization in non-infected and infected JAWS-II DCs. [file Image_8.tif]
